# Development of a real-time RT-PCR and Reverse Line probe Hybridisation assay for the routine detection and genotyping of Noroviruses in Ireland

**DOI:** 10.1186/1743-422X-4-86

**Published:** 2007-09-06

**Authors:** John F Menton, Karen Kearney, John G Morgan

**Affiliations:** 1 Lab 439, Food Science Building, Department of Microbiology, University College Cork, Cork, Republic of Ireland

## Abstract

**Background:**

Noroviruses are the most common cause of non-bacterial gastroenteritis. Improved detection methods have seen a large increase in the number of human NoV genotypes in the last ten years. The objective of this study was to develop a fast method to detect, quantify and genotype positive NoV samples from Irish hospitals.

**Results:**

A real-time RT-PCR assay and a Reverse Line Blot Hybridisation assay were developed based on the ORF1-ORF2 region. The sensitivity and reactivity of the two assays used was validated using a reference stool panel containing 14 NoV genotypes. The assays were then used to investigate two outbreaks of gastroenteritis in two Irish hospitals. 56 samples were screened for NoV using a real-time RT-PCR assay and 26 samples were found to be positive. Genotyping of these positive samples found that all positives belonged to the GII/4 variant of NoV.

**Conclusion:**

The combination of the Real-time assay and the reverse line blot hybridisation assay provided a fast and accurate method to investigate a NoV associated outbreak. It was concluded that the predominant genotype circulating in these Irish hospitals was GII/4 which has been associated with the majority of NoV outbreaks worldwide. The assays developed in this study are useful tools for investigating NoV infection.

## Background

Noroviruses (NoV) are one of the most common causes of acute non-bacterial gastroenteritis in humans. Formerly known as "Norwalk virus", NoV was first recognised in October 1968 in an elementary school in Norwalk, Ohio [1]. It is highly contagious and can be transmitted as an aerosol, through direct contact or *via *the faecal oral route causing explosive outbreaks in environments where people are in close contact such as hospitals, retirement homes, cruise ships, army bases, hotels and holiday resorts [2-5]. Symptoms consist of severe vomiting and diarrhoea which can last for 24–72 hours. NoV is a non-enveloped virus with a capsid of 27–35 nm in diameter and is a member of the *Calicivirus *family. The virion consists of a single positive strand RNA genome of approximately 7.6 kb and encodes three open reading frames. ORF1 encodes the nonstructural proteins, ORF2 encodes the major capsid protein VP1 and ORF3 encodes a minor structural protein VP2.

The emergence of NoV as the most prominent cause of gastroenteritis over the last ten years is due to improved methods of detection, which have allowed accurate identification of the viruses responsible for these outbreaks. The most utilised methods are Electron Microscopy (E.M) and Reverse Transcription Polymerase Chain Reaction (RT-PCR). RT-PCR is the preferred method as it is rapid and very sensitive; however, it relies heavily on precise primer design which can be problematic due to the high level of genetic variability between NoV strains. Human NoV can be divided into three Genogroups GI, GII and GIV which can be further subdivided into 8, 17 and 1 genotypes respectively based on VP1 sequences [6].

The use of a broadly reactive primer pairs allows accurate detection of NoV, however typing the strain of NoV responsible for an outbreak still relies heavily on sequencing, which can be time consuming

In this study, we describe a real-time RT-PCR assay based on SYBR Green chemistry utilising a broadly reactive pair of primers for both GI and GII NoV based on the highly conserved ORF1-ORF2 junction. A reverse line blot hybridisation assay was developed within this ORF1-ORF2 junction by designing 25 genotype specific probes to allow rapid detection and typing of an outbreak. This method was used to detect and genotype virus present in the stools of patients suffering from gastroenteritis in two outbreaks which occurred in Irish Hospitals in 2005 and 2006.

## Results

### Development and validation of a SYBR green based Real-Time RT-PCR assay for NoV

Two degenerate reverse primers G1NVR and G2NVR (Table 1) were designed based on multiple alignments of 30 Genogroup I sequences and 120 Genogroup II sequences. The sequences were 400 bp segments of the ORF1-ORF2 junction (region 5288–5665 nts Southampton and region 5005–5387 nts Lordsdale). These primers were combined with previously published primers designed by Kageyama et al., 2003 [7] to detect human NoV.

**Table 1:** Primers and probes used for Lightcycler RT-PCR and Reverse Line Blot hybridization assay.

Primers	Primer Sequence	Reference
COG1F	CGY TGG ATG CGN TTY CAT GA	[7]
G1NVR	ACC CAR CCA TTA TAC ATY TG	
		
COG2F	CAR GAR BCN ATG TTY AGR TGG ATG AG	[7]
G2NVR	ACC NGC ATA NCC RTT RTA CAT TC	
		
Probes		
		
GI/1	TCT TGC AAT GGA TCC TGT RGC RG	
GI/2	GAA CCC GTG GCY GGG CCA AC	
GI/3	CCA GAG GCA AAY ACA GCT GAG	
GI/4	TGA CCC TGT GGC TGG CTC CTC	
GI/5	ATG CTG AAC CAC TGC CWC TTG AT	
GI/6	CAA ATT TCA ATG GAY CCT GTT GCG	
GI/7	GGT AGT GGG CGC CGC AAC C	
GI/8	TGC GGT TGC TAC TGC CGG CCA	
		
GII/1	CGA GAC GAT GGC MCT CGA ACC G	
GII/2	TAT AGA CCC TTG GAT TAG AGC A	
GII/3	CAA TGG CGC TAG ABC CAG TGG CG	
GII/4	GAC GCC AAC CCA TCT GAT GGG TC	
GII/5	GGT GGG GGC GTC TTT AGC C	
GII/6	CTC AAT CGC AGC TCC TGT YGT	
GII/7	GCA TCG CTG GCG ACA CCA GTT G	
GII/8	TCA ACC ATG AGG TCA TGG CCA TA	
GII/9	CCC CGG GTG AGT TCT TGC TYG A	
GII/10	TTC CCC TGG AGA AGT ACT CCT	
GII/12	CGA ACT AAA TCC ATA CCT AGC ACAC	
GII/13	CAG TGG CGG GAC AAA CCA AC	
GII/14	CTC TCC TGG AGA ACT CCT ACT TGA T	
GII/15	GAA GTC TTG CCT TTA GAG CCC GTC	
GII/16	CAG TTG CGG GAG CTT CAA TCG CT	
GII/17	CCT CCC TTT GGA ACC AGT TGC	

**Figure 1:**
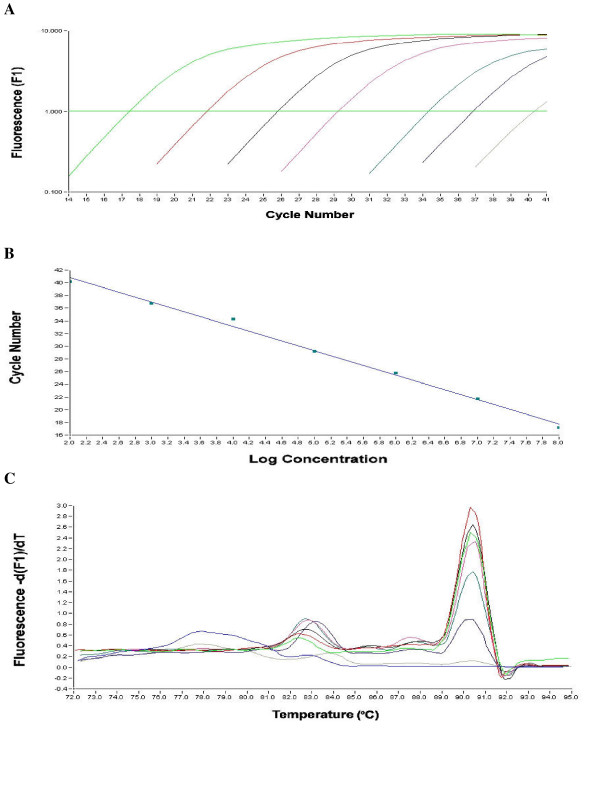
(A) Amplification of standards showing fluorescence versus cycle number concentration of 10^7 ^– 10^1 ^molecules are shown from left to right. (B) Standard curve of GI assay R_2 _1.00 and a slope of -3.8 was obtained. (C) Melting curve of GI standards showing melting point at 90°C in descending order 10^7 ^– 10^1 ^molecules.

**Figure 2:**
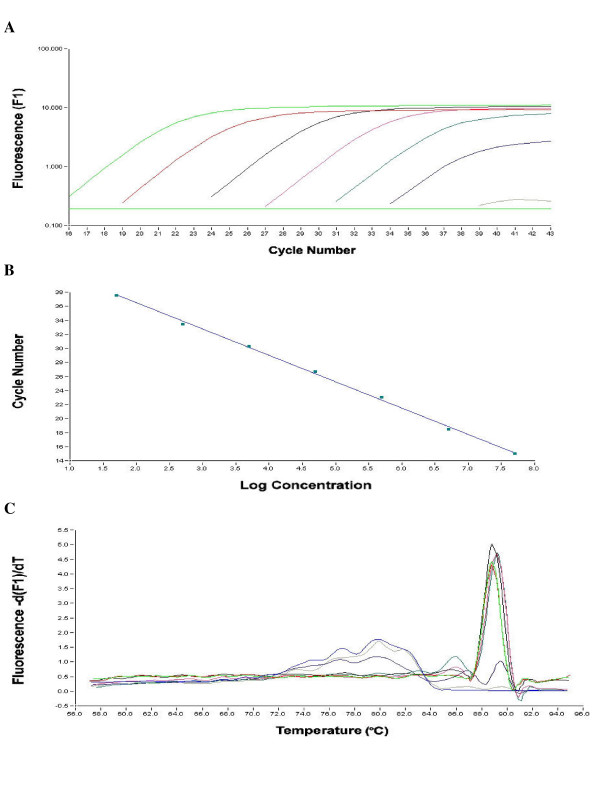
(A) Amplification of GII standards showing fluorescence versus cycle number concentration of 5 × 10^7 ^– 5 × 10^1 ^molecules are shown from left to right. (B) Standard curve of GII assay R_2 _1.00 and a slope of -3.7 was obtained. (C) Melting curve of GII standards showing melting point at 88°C in descending order 5 × 10^7 ^– 5 × 10^1 ^molecules.

Two separate Real-time RT-PCR assays were designed based on SYBR green chemistry for GI and GII NoV. This assay utilised a fluorescence acquisition step at 85°C for GI and 84°C for GII to melt primer dimers thus ensuring only amplified product was detected. Standard curves were created in triplicate using serial dilutions of plasmids containing GI/2 and GII/4 PCR products corresponding to Southampton virus and Oxford B2S16. Detection levels of these plasmid molecules were 10^7 ^to 10^1 ^for GI and 5 × 10^7 ^to 5 × 10^1 ^for GII (Fig. 1A and Fig. 2A). Melting curve analysis identified positive samples by large peaks at ~90°C and ~88°C respectively for GI and GII NoV (Fig. 1C and Fig. 2C). A stool panel containing 5 genotypes of GI NoV and 9 genotypes of GII NoV was obtained from external laboratories (Table 2). This stool panel was applied to the Lightcycler assay and all the genotypes were detectable.

**Table 2:** Stool panel acquired by this group 2003–2005.

Norovirus panel	Norovirus strain	Source
Genogroup I		
GI/I	Hu/NoV/West Chester/2001/USA	CDC, USA
GI/2	Hu/NV/SHV/1993UK	UK
GI/3	Hu/NV/Stav/1999/Nor	Irish isolate
GI/4	Hu/Nv/Saitama T69GI/2002/JP	CDC, USA
GI/6	Hu/Nv/Saitama T44GI/2001/JP	CDC, USA
Genogroup II		
GII/2	Hu/NoV/Melksham/2001/USA	CDC, USA
GII/3	Hu/NoV/VannesL169/2000/France	HPA, UK
GII/4	Hu/NLV/GII/Carlow/2002/Irl	Irish isolate
GII/6	Hu/NoV/SU4-JPN/2002/JP	CDC, USA
GII/8	Hu/NoV/Saitama T67GII/2002/JP	HPA, UK
GII/10	Hu/NoV/Mc37/2004/JP	CDC, USA
GII/12	Hu/NoV/Honolulu/314/1994/US	CDC, USA
GII/16	Hu/NoV/Hiram/2000/USA	CDC, USA
GII/17	Hu/NoV/CS-E1/2002/USA	CDC, USA

CDC : Centres for Disease control

HPA: Health Protection agency

### Detection and quantification of human NoV by Real-Time RT-PCR

56 samples were taken from two outbreaks of NoV in two Irish hospitals in 2005 and 2006. These samples were applied to the GII NoV real-time assay and 26 samples were detected as positive for GII NoV. Samples negative for GII NoV were applied to the GI assay and were also found to be negative for GI NoV. Samples were quantified using the plasmid standard curve. The lowest Ct value was at point 35.79, giving a concentration of 2.67 × 10^2 ^molecules of NoV cDNA or 2.67 × 10^6 ^per gram of stool. The highest Ct value was at point 21.93 giving a concentration of 7.53 × 10^5 ^molecules of NoV cDNA or 7.53 × 10^9 ^molecules per gram of stool. The average number of NoV molecules per gram of stool was 1.02 × 10^9^molecules.

### Design of oligonucleotide probes for development of reverse line probe hybridization assay

Twenty-five oligonucleotide probes were designed within the region of the COG1F-G1NVR and COG2F-G2NVR PCR products. Design was based on a annealing temperature of at least 60°C and a minimum of 3 mismatches between the probe and the other genotypes. All the probes were submitted to BLASTn program National Centre for Biotechnology Information to verify specificity. A probe was designed for each genotype within GI (1–8) and GII (1–17) NoV classified according to Zheng et al., 2006 [6]. The GII/11 probe was excluded from the membrane as this genotype has only been associated with porcine NoV.

**Figure 3:**
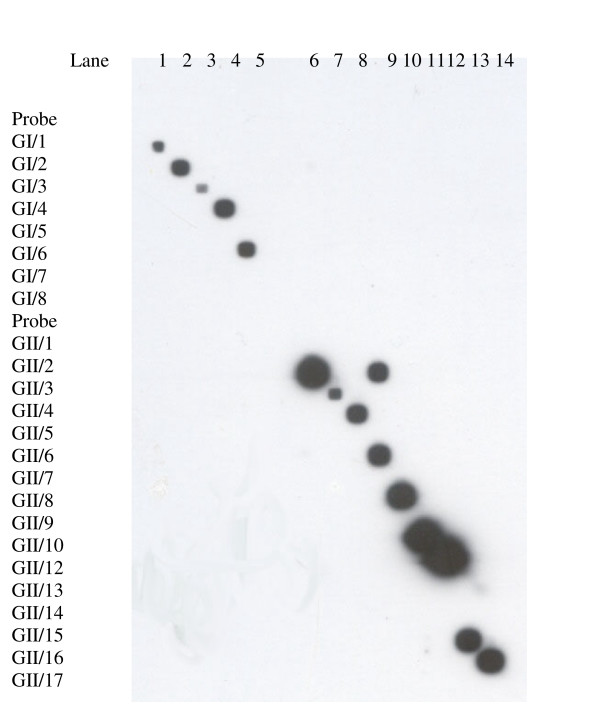
**Validation of Reverse Line Blot hybridization using stool panel samples. **Left of diagram indicates where the 25 probes are fixed across the membrane Top of the figure indicates where denatured PCR products of stool panel have been applied. Presence of spot indicates probe binding. Gaps between spots indicate unbound probes for which no reference samples are available. Lane 1 : GI/1, 2 : GI/2, 3 : GI/3, 4 : GI/4, 5 : GI/6, 6 : GII/2, 7 : GII/3, 9 : GII/6, 10 : GII/8, 11 : GII/10, 12 : GII/12, 13 : GII/16, 14 : GII/17.

Probes were covalently bound to a negatively charged nylon membrane and the membrane was rotated 90° horizontally. Denatured PCR products of all 14 positive samples in the NoV panel were annealed to the membrane giving specific binding i.e. single dots were observed for all respective probes with the exception of probe GII/2 which binds both GII/2 and GII/6 (Fig. 3).

### Genotyping of NoV positive samples

**Figure 4:**
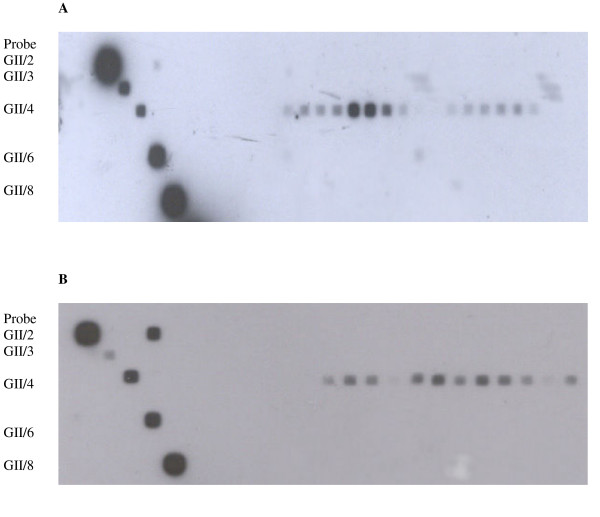
Genotyping of the (A) 14 positive samples from the 2005 outbreak and (B) 12 positive samples from the 2006 outbreak. Control samples containing known RT-PCR products from the reference panel are shown on the left binding to there respective probes. Clinical samples binding to probe GII/4 can be seen on the right of both A and B.

26 positive samples from the two outbreaks were applied to the membrane along with 9 GII samples from the stool panel. The 12 RT-PCR positive samples from the 2006 outbreak and the 14 positive samples from the 2005 outbreak all bound the GII/4 probe (Fig. 4A and 4B).

## Discussion

The reverse primers designed in this study were combined with the forward primers designed by Kageyama et al., 2003 [7] to create a conventional RT-PCR assay for human NoV. These primer pairs compared favourably (data not shown) with three previously published RT-PCR assays [7-9]. A Real-Time SYBR green RT-PCR assay was then developed based on the primer pairs to detect and quantify both GI and GII NoV in the Irish population. The chemistry of SYBR green allows non-specific products such as primer dimers to yield a fluorescent signal. This was overcome by incorporating a fluorescent read step at 85°C for the GI assay and 84°C for the GII assay. This adjustment means that only NoV RT-PCR product is measured by the Real-Time thermocycler.

The assay demonstrated good sensitivity, detecting from 10^7 ^to 10^1 ^molecules of plasmid DNA for GI NoV and 5 × 10^7 ^to 5 × 10^1 ^for GII NoV. The R_2 _values for both standard curves were 1.00 with a slope of -3.5 and -3.7 respectively for GI and GII NoV (Fig. 1B and Fig. 2B). Melting curve analysis showed a positive peak at ~90°C and 88°C for GI and GII NoV. The broad reactivity of the assay was validated using a panel of stool samples collected containing 5 GI and 9 GII NoV genotypes. The GI based NoV assay detected all five of the different genotypes of GI stool panel (GI/1, GI/2, GI/3, GI/4 and GI/6) and showed no cross reactivity with any of the GII NoV. The GII assay detected all 9 Genotypes of the Genogroup II stool panel (GII/2, GII/3, GII/4, GII/6, GII/8, GII/10, GII/12, GII/16, GII/17) and showed no cross reactivity with GI NoV.

It was not possible to obtain all of the NoV genotypes to validate the assay described. GI/5, GI/7 and GI/8 were not available for Genogroup I and GII/1, GII/5, GII/7, GII/9, GII/13, GII/14, GII/15 were not available for Genogroup II. However, the fact that a large range of the genotypes were successfully amplified i.e. GI/1 to GI/6 and GII/2 to GII/17, coupled with the evidence based on multiple alignments of current sequence data available for NoV (data not shown) indicates that the RT-PCR assay described here would be appropriate as a broad range detection method for NoV infection.

The real-time assay was used to detect and quantify the presence of 26 NoV positive samples from 56 samples obtained from two Irish hospitals. The average titre of virus per gram of stool was found to be 1.02 × 10^9 ^molecules with the range of titre running from 2.67 × 10^6 ^per gram of stool to 7.53 × 10^9 ^molecules per gram of stool. These high levels are consistent with numbers reported in other studies of NoV levels in stools [7,10].

The basis of this quantification was on assumption of 100% RT efficiency. This method allows a calculation of the minimum amount of NoV present based on cDNA values. Ideally quantification of an RNA virus involves the use of an RNA standard. However, RNA standards are not very stable, thus making standard curve construction difficult. It is more practical to use plasmids for the construction of an external standard curve. A recommendation to this problem would be the generation of an armoured RNA control for both GI and GII NoV similar to those available for Hepatitis C [11].

The genotyping of positive NoV obtained from outbreaks is usually performed by direct sequencing of the PCR products, a time consuming process. A first generation line-probe assay was created for genotyping NoV based on the highly conserved ORF1-ORF2 region. The primer pair described in this paper contains sufficient sequence variability between the primer binding sites to allow the design of specific probes for each genotype. The assay was validated using the stool panel of 14 different NoV genotypes. It was found that at an annealing temperature of 57°C, both the GI and GII probes bound specifically to their genotypes present in the panel with the exception of GII/2 which also binds GII/6 (Fig. 3). Analysis of multiple sequences of GII/2 revealed that it was not possible to design a probe which would not bind GII/6. Therefore, it is not possible using this assay to differentiate GII/2 and GII/6 in an unknown sample.

No cross reactivity was observed between the probes GI/5, GI/7, GI/8 GII/1, GII/5, GII/7, GII/9, GII/13, GII/14, GII/15 with the genotypes present in the stool panel (Fig. 3). The lack of cross reactivity allows these probes to be left on the membrane and as they are designed to anneal to PCR products at the same temperature as the validated probes they should detect their corresponding genotypes in unknown samples.

Applying this assay to the 14 positives from the 2005 outbreak and the 12 positives from the 2006 outbreak (Fig. 4A and 4B) detected by Real-time RT-PCR revealed that all 26 of the samples were genotype GII/4. This result was confirmed by sequencing of the RT-PCR products. The predominance of the GII/4 genotype is consistent with previous sequence analysis of Irish NoV isolates [12,13] and with that of other NoV circulating globally [14-17].

This paper describes both a rapid, sensitive and broadly reactive method for detecting and genotyping Human NoV. As the same sets of primers are used for both assays the combination of both methods greatly speeds up ascertaining when a NoV outbreak is occurring and which strain is responsible. A SYBR green mastermix containing a proof reading enzyme would again speed up the typing process as this would allow the RT-PCR products to be applied directly to the membrane by allowing biotin primers to be used in the initial detection of NoV. Assays like these have been developed for Human papillomavrius [18]. The genotyping assay would also be useful for investigating outbreaks associated with water or oysters as it would allow typing of possible mixed infections which may occur due to the nature of both these contaminants. Future development of this assay would involve developing a disposable Line-probe assay based on these probes similar to those commercially available combined with automation to further improve the timeframe for genotyping a NoV infection.

## Conclusion

A real time RT-PCR assay and a RLBH assay were developed and utilised to identify and genotype the causative agent of two gastroenteritis outbreaks in two Irish hospitals. The amount of Nov present in infected stool samples was estimated and the strain of NoV responsible for all positive cases was genotyped as the GII/4 variant.

## Methods

### Clinical specimens

A reference panel of various genotypes of NoV was acquired between January 2003 and December 2006 to determine the broad reactivity of the primers and probes. The panel was transported on dry ice and remained frozen at -20°C until processing This panel was screened by RT-PCR and the products were TA cloned (Invitrogen) (Table 2). Fifty six stool samples were collected from both Waterford Regional hospital and the Mercy Hospital Cork from January 2004 to March 2006 and stored at 4°C prior to processing.

### Primer and probe design

A multiple alignment was performed using the MEGALIGN programme (DNASTAR). Thirty sequences of GI and 120 sequences of GII were aligned using this program. COG1F and COG2F primers described by [7] were chosen as forward primers for GI and GII assays respectively. Two reverse primers were designed based on these alignments and denoted G1NVR and G2NVR (Table 1).

Eight oligonucleotide probes were designed for the detection of GI NoV and 17 probes were designed for the detection of GII NoV. The probes were designed based on the criteria that they were at least 20 nucleotides in length and that they had a Tm of at least 60°C. The probes were 5' hexylamine labelled (Operon, Germany).

### Extraction of viral RNA

Stools were diluted in a 10% (w/v) Modified Eagles Medium (Gibco). The suspension was centrifuged at 10000 rpm for 10 min and 200 μl supernatant was applied to the High Pure Viral nucleic extraction kit (Roche). The extracted RNA was DNAse treated using RNAse free DNAse (Ambion).

### Reverse Transcription (RT)

RT was performed using a Superscript II Reverse transcriptase kit (Invitrogen™) to a final volume of 20 μl. 10 μl of extracted RNA and 1 μl of 75 pmole random hexamers (Roche) are added to a 0.5 ml PCR reaction tube, mixed and heated to 95°C for 3 min. A master-mix was prepared according to the manufacturer's instructions and incubated as directed.

### Detection of Norovirus

NoV was detected by a Lightcycler assay (Roche Applied Science) designed in our laboratory based on the COG1F-GINVR or COG2F-G2NVR primers (Table 1). Quantitative RT-PCR was performed using the LightCycler^® ^FastStart DNA Master SYBR Green I (Roche). A final reaction volume of 20 μl containing 0.5 μl of cDNA, 2 μl of SYBR green Mastermix, 2.8 μl of 25 mM MgCl_2 _(3.5 mM), 1 μl of each primer (0.6 μM) and 12.7 μl of PCR grade water. The reaction was performed using GI primers with a denaturation step of 94°C for 8 min followed by 40 cycles at 94°C for 10 s, 45°C for 10 s 72°C for 15 s and a fluorescent read step of 85°C for 15 s to melt primer dimers.

The reaction was performed using GII primers with a denaturation step of 94°C for 8 min followed by 45 cycles of 94°C for 5 s, 52°C for 10 s, 72°C for 17 s and a fluorescent read step of 84°C for 10 s to melt primer dimers. For the creation of standard curves, 2 μl containing dilutions of 10^7 ^to 10^1 ^molecules of GI/2 plasmid or 5 × 10^7 ^to 5 × 10^1 ^molecules of GII/4 plasmid DNA were added to the reaction tubes. All reactions were run with negative controls and subjected to melting curve analysis.

### Biotinylated RT-PCR

Biotinylated reverse primers G1NVR and G2NVR synthesized by MWG Biotech (Ebersberg, Germany) were used in the following RT-PCR assay at a final volume of 50 μl. The reaction contained 4 μl of cDNA from the RT reaction, 5 μl of 10× PCR Buffer, 1.5 mM MgCl_2_, 1 μl of 10 mM each of dATP, dCTP, dGTP, and dTTP per reaction, 1 μM each of primers and 2.5 units of Platinum Taq polymerase (Invitrogen) was performed on a MJ PTC-200 thermocycler (MJ research). The reaction was performed for GI with a denaturation step at 94°C for 3 min, followed by 40 cycles at 94°C for 1 min, 45°C for 1 min, 68°C for 1 min and a final extension at 68°C for 7 min. The PCR for GII was a denaturation at 94°C for 3 min, followed by 40 cycles at 94°C for 30 s, 48°C for 30 s, 72°C for 1 min and a final extension at 72°C for 7 min. The PCR products were separated on a 2% agarose gel and visualized by ethidium bromide staining. The PCR products for Genogroup I and Genogroup II were 387 bp and 378 bp in length, respectively.

### Reverse Line Blot Hybridization

Twenty-five oligonucleotide probes each corresponding to a GI or GII genotype (Table 2) which were synthesized with a 5' hexylamino group (Operon Biotechnologies Ltd, Cologne, Germany). The oligonucleotides were covalently bound to a negatively charged nylon membrane (Biodyne C; Pall Biosupport, Portsmouth, Cambridge, United Kingdom) by this 5' hexylamino group. Briefly, the carboxyl groups on the membrane were activated by incubation for 10 min in 16% (w/v) 1-ethyl-3-(3-dimethylaminopropyl) carbodimide (EDAC) (Sigma). The membrane was washed with tap water and placed in a miniblotter system (MN45; Immunetics, Cambridge, Massachusetts). The slots were filled in parallel with 150 μl of each of the 5'-hexylamine-labeled oligonucleotides at a final concentration of 1 μM diluted in freshly prepared 0.5 M NaHCO3 [pH 8.4] and after 1 min of incubation at room temperature, the excess solution was aspirated and the membrane was removed from the miniblotter. The remaining active esters on the membrane were hydrolyzed by incubation in 0.1 M NaOH for 8 min at room temperature and rinsed in water. The membrane was washed twice for 5 min at 60°C in 2 × SSPE (Sigma) with 0.1% sodium dodecylsulfate (SDS) (BDH, Poole, United Kingdom). The membrane was used immediately or washed for 15 min in 20 mM EDTA and stored sealed in plastic at 4°C.

Prior to use in hybridization, the membrane was washed for 5 min in 2 × SSPE-0.1% SDS, placed in the miniblotter. The membrane was rotated so that the probes were perpendicular to the previous position. 15 μl of each PCR product in 135 μl of 2 × SSPE-0.1% SDS was denatured by heating to 99°C for 10 min and chilled on ice. The slots were then filled with 150 μl of PCR product and incubated for 60 min at 57°C in a hybridization oven. After hybridization, unbound PCR product was removed by washing twice with prewarmed 2 × SSPE-0.5% SDS at 60°C for 10 min. The membrane was then incubated at 42°C in 10 ml of 1:2000 dilution of streptavidin-peroxidase conjugate (Roche) in prewarmed 2 × SSPE buffer for 1 hr. Unbound streptavidin-conjugate was removed by washing twice with 2 × SSPE-0.5% SDS at 42°C for 10 min. lastly the membrane was washed twice with 2 × SSPE at room temperature for 5 min to remove SDS.

The bound PCR products were detected by a chemiluminescence assay using ECL detection liquid (Roche) and visualized by exposure of the blot for 10 min to 3 hrs to an X-ray film (Hyperfilm; Amersham). For repeated use, the membranes were stripped twice in 1% SDS at 80°C for 30 min and incubated for 15 min at room temperature in 20 mM EDTA solution, the membranes were sealed and stored at 4°C until further use.

## Abbreviations

Norovirus (NoV); Reverse transcription PCR (RT-PCR); Genogroup I (GI); Genogroup II (GII); Reverse Line Blot Hybridisation (RLBH).

## Competing interests

The author(s) declare that they have no competing interests.

## Authors' contributions

JFM is the corresponding author and main contributing author of this manuscript. KK contributed to primer design, the construction of the plasmids used for standard curve generation and the final review of the manuscript. Supervision and final review of the manuscript was provided by JGM. All authors have read and approved the final manuscript.

## References

[B1] Kapikian AZ (2000). The discovery of the 27-nm Norwalk virus: an historic perspective. J Infect Dis.

[B2] Saito H (2002). [Epidemiology on Norwalk virus-related gastroenteritis outbreaks among elderly persons living in nursing homes]. Nippon Rinsho.

[B3] Gallimore CI, Richards AF, Gray JJ (2003). Molecular diversity of noroviruses associated with outbreaks on cruise ships: comparison with strains circulating within the UK. Commun Dis Public Health.

[B4] Blanton LH, Adams SM, Beard RS, Wei G, Bulens SN, Widdowson MA, Glass RI, Monroe SS (2006). Molecular and epidemiologic trends of caliciviruses associated with outbreaks of acute gastroenteritis in the United States, 2000-2004. J Infect Dis.

[B5] Lang L (2003). Acute gastroenteritis outbreaks on cruise ships linked to Norwalk-like viruses. Gastroenterology.

[B6] Zheng DP, Ando T, Fankhauser RL, Beard RS, Glass RI, Monroe SS (2006). Norovirus classification and proposed strain nomenclature. Virology.

[B7] Kageyama T, Kojima S, Shinohara M, Uchida K, Fukushi S, Hoshino FB, Takeda N, Katayama K (2003). Broadly reactive and highly sensitive assay for Norwalk-like viruses based on real-time quantitative reverse transcription-PCR. J Clin Microbiol.

[B8] Kojima S, Kageyama T, Fukushi S, Hoshino FB, Shinohara M, Uchida K, Natori K, Takeda N, Katayama K (2002). Genogroup-specific PCR primers for detection of Norwalk-like viruses. J Virol Methods.

[B9] O'Neill HJ, McCaughey C, Wyatt DE, Mitchell F, Coyle PV (2001). Gastroenteritis outbreaks associated with Norwalk-like viruses and their investigation by nested RT-PCR. BMC Microbiol.

[B10] Pang X, Lee B, Chui L, Preiksaitis JK, Monroe SS (2004). Evaluation and validation of real-time reverse transcription-pcr assay using the LightCycler system for detection and quantitation of norovirus. J Clin Microbiol.

[B11] WalkerPeach CR, Winkler M, DuBois DB, Pasloske BL (1999). Ribonuclease-resistant RNA controls (Armored RNA) for reverse transcription-PCR, branched DNA, and genotyping assays for hepatitis C virus. Clin Chem.

[B12] Waters A, Coughlan S, Dunford L, Hall WW (2006). Molecular epidemiology of norovirus strains circulating in Ireland from 2003 to 2004. Epidemiol Infect.

[B13] Foley B, O'Mahony J, Hill C, Morgan JG (2001). Molecular detection and sequencing of "Norwalk-like viruses" in outbreaks and sporadic cases of gastroenteritis in Ireland. J Med Virol.

[B14] Lynch M, Painter J, Woodruff R, Braden C (2006). Surveillance for foodborne-disease outbreaks--United States, 1998-2002. MMWR Surveill Summ.

[B15] Vainio K, Myrmel M (2006). Molecular epidemiology of norovirus outbreaks in Norway during 2000 to 2005 and comparison of four norovirus real-time reverse transcriptase PCR assays. J Clin Microbiol.

[B16] Koopmans M, Harris J, Verhoef L, Depoortere E, Takkinen J, Coulombier D (2006). European investigation into recent norovirus outbreaks on cruise ships: update. Euro Surveill.

[B17] Kearney K, Menton J, Morgan JG (2007). Carlow Virus, a 2002 GII.4 variant Norovirus strain from Ireland. Virol J.

[B18] Payan C, Ducancelle A, Aboubaker MH, Caer J, Tapia M, Chauvin A, Peyronnet D, Le Hen E, Arab Z, Legrand MC, Tran A, Postec E, Tourmen F, Avenel M, Malbois C, De Brux MA, Descamps P, Lunel F (2007). Human papillomavirus quantification in urine and cervical samples by using the Mx4000 and LightCycler general real-time PCR systems. J Clin Microbiol.

